# Age-Stratified Spatial Radiological Risk Assessment of ^226^Ra ^232^Th and ^40^K in Water Surrounding the Geita Gold Mine in Tanzania

**DOI:** 10.3390/jox15050152

**Published:** 2025-09-16

**Authors:** Jerome M. Mwimanzi, Nils H. Haneklaus, Farida Lolila, Janeth J. Marwa, Mwemezi J. Rwiza, Kelvin M. Mtei

**Affiliations:** 1 School of Materials Energy Water and Environmental Sciences, Nelson Mandela African Institution of Science and Technology, Arusha P.O. Box 442, Tanzania; 2 Tanzania Atomic Energy Commission, Arusha P.O. Box 743, Tanzania; 3 Td-Lab Sustainable Mineral Resources, Universität für Weiterbildung Krems, Dr.-Karl-Dorrek-Straße 30, 3500 Krems an der Donau, Austria; 4 Unit for Energy and Technology Systems—Nuclear Engineering, North-West University, 11 Hoffman Street, Potchefstroom 2520, South Africa; 5 Faculty of Science, Dar es Salaam University College of Education, Dar es Salaam P.O. Box 2329, Tanzania; 6 School of Business Studies and Humanities, Nelson Mandela African Institution of Science and Technology, Arusha P.O. Box 442, Tanzania

**Keywords:** ingestion dose, natural radioactivity, radiological risk assessment, radioactive material, water quality monitoring, Geita Gold Mine

## Abstract

Long-term ingestion of water contaminated with naturally occurring radioactive material (NORM) may pose health risks. Water around the Geita Gold Mine in Tanzania was assessed by high-purity germanium gamma spectrometry to quantify the activity concentrations of ^226^Ra, ^232^Th, and ^40^K, and computed age-stratified ingestion doses and risk indices were determined. The average activity concentrations were 57 mBq L^−1^ for ^226^Ra and 5026 mBq L^−1^ for ^40^K, while the activity concentrations of ^232^Th were below the detection limit in all samples. The estimated adult fatal cancer risk ranged from 0.9 × 10^−6^ to 3.1 × 10^−6^ (mean 2.0 × 10^−6^). The excess lifetime hereditary effect ranged from 2.0 × 10^−6^ to 7.3 × 10^−6^ for males (average 4.5 × 10^−6^ ± 1.5 × 10^−6^) and 2.1 × 10^−6^ to 7.7 × 10^−6^ for females (average 4.8 × 10^−6^ ± 1.6 × 10^−6^). One-way ANOVA and Pearson correlations indicated significant spatial variation in activities and indices across sites and age groups. Under current conditions, waters appear to be radiologically safe. However, mine-adjacent hotspots warrant targeted surveillance. The obtained results provide a baseline for sound monitoring approaches at the Geita Gold Mine and other mines showing similar activity profiles.

## 1. Introduction

Human exposure to ionizing radiation is predominantly due to naturally occurring radioactive material (NORM), which account for approximately 85% of the total exposures [1,2]. Cosmic rays also provide a contribution of approximately 15% to radiation doses that are “naturally occurring”, but non-terrestrial. NORM, including Potassium-40 (^40^K) as well as the decay series of Uranium-238 (^238^U), Uranium-235 (^235^U), Uranium-234 (^234^U) and Thorium-232 (^232^Th), occur ubiquitously in the Earth’s crust at trace levels [3]. However, anthropogenic activities, such as mining for uranium, but also for other elements such as gold, where ore bodies show trace amounts of NORM, can lead to the mobilization of these radionuclides into the environment. Water can act as a transport medium for liberated radionuclides through pathways such as groundwater pumped from underground mine process waters, storm-water runoff from waste rock dumps, and tailings storage facilities, as well as seepage and effluents from tailings dams and retention ponds [4].

Once mobilized into water, alpha-, beta- and gamma-emitting radionuclides such as ^226^Ra and its decay products maybe ingested by the local population, posing cumulative radiological hazards over years to decades [3,5]. Typically, ^226^Ra is in secular equilibrium with its decay products (until disturbed), whereas ^40^K is an environmentally mobile radionuclide. The ^226^Ra radionuclide mimics calcium biochemistry, concentrating in bone marrow and irradiating it, while ^40^K, through an essential element, is absorbed like potassium and distributed throughout the body [5].

Global surveys have documented elevated radionuclide activity and ingestion doses near uranium and gold mines. The reported activities often exceed the recommended individual dose of 100 µSv y^−1^ [6]. For instance, South African gold fields have reported annual ^226^Ra ingestion doses exceeding 200 µSv y^−1^ [7,8].

Tanzania applies a dose limit of 1 mSv y^−1^ for internal doses (ingestion/inhalation), which is in line with IAEA GSR Part 3 [9]. For drinking water, results are judged against a 0.1 mSv y^−1^ screening reference for the public, alongside radionuclide-specific screening levels of 1 Bq L^−1^ and 10 Bq L^−1^ for ^226^Ra and ^40^K [10,11,12]. Despite several studies reporting elevated levels of trace elements and heavy metals from water sources, soil, sediments and plants near gold mining areas in Tanzania [13,14,15,16,17], radiological analysis of nearby water is scarce. By contrast, assessments in Tanzania have largely focused on uranium mining sites and the Minjingu phosphate deposit that contains elevated levels of uranium [18,19,20]. For instance, higher activity concentrations in ground water were reported at the Mkuju River Uranium Project, and elevated NORM near the Bahi uranium deposits (with ^226^Ra and ^232^Th above global averages) has been reported, while other sites showed comparatively low activities [10,11,12]. To our knowledge, no prior study has provided an age-stratified spatially resolved radioanalytical assessment for a Tanzanian gold mine. This study quantifies the activity concentrations of ^226^Ra, ^232^Th, and ^40^K in water surrounding the Geita Gold Mine using gamma spectrometry, models ingestion doses for infants children and adults, employs spatial analysis with one-way ANOVA to identify radiological contamination hotspots, and evaluates the influence of water quality parameters such as pH, electrical conductivity (EC), and total dissolved solids (TDS) on NORM mobilization.

## 2. Materials and Methods

### 2.1. Description of Study Area

**Figure 1:**
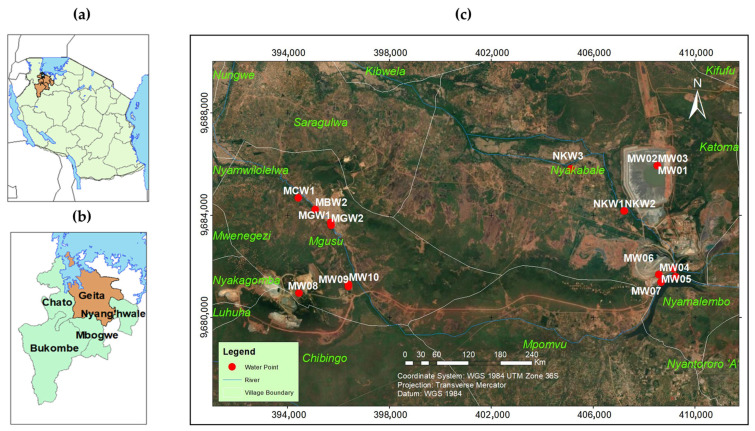
Location of the study area and sampling points. (**a**) Map of Tanzania showing the location of the Geita region. (**b**) Administrative boundaries of the Geita district. (**c**) Imagery of the study and sampling points.

The study was carried out at the Geita Gold Mine and the villages which surround the mine (Figure 1). The Geita Gold Mine is one of the largest and longest-running gold mines in Tanzania, located in northwestern Tanzania, within the Lake Victoria goldfields, a region renowned for its rich mineral resources. The geological formation of the study area consists of banded iron formations (BIFs), felsic volcanic, and andesite/diorite lithology, which may contribute to radiological risks [21,22]. The mine is surrounded by six predominantly rural villages, where livelihoods primarily rely on subsistence and small-scale farming, as well as livestock keeping. Additionally, the mine’s proximity encourages informal artisanal mining and trade activities.

### 2.2. Sample Collection and Analysis

Sampling sites were selected to represent a gradient of exposure conditions ranging from proximity to tailings, local hydrology, and population water usage patterns, following the protocol in the study by Ameho, et al. [23]. This ensured comprehensive spatial coverage, including spring wells, ponds, tailings dams, and rivers. As such, water was collected in pre-cleaned high-density polyethylene (HDPE) thoroughly rinsed with deionized water. Each 1 L aliquot was acidified with 1.0 mL of 2 M HNO_3_ acid to prevent radionuclide adsorption onto container walls. Moreover, in situ measurements of pH, electrical conductivity (EC), and total dissolved substances (TDS) were performed using a Horiba U-51 multi-parameter meter (Horiba Ltd., Kyoto, Japan), which is equipped with combined electrodes [24]. The instrument was calibrated using a three-point procedure with standard buffer solutions at pH 4.00, 7.00, and 10.00. Calibration accuracy was verified by cross-checking with the same standards to confirm proper sensor response across the operational range for pH.

Following the field measurements, water samples were transported to the Tanzania Atomic Energy Commission (TAEC) laboratory. At the TAEC laboratory, each water sample was kept in a sealed and airtight 1 L Marinelli beaker to prevent loss of ^222^Rn gas. Then, the samples were stored for 30 days to allow ^222^Rn and its progeny to reach secular equilibrium prior to gamma spectrometric analysis [23,25].

### 2.3. Radiometric Analysis

The activity concentrations of the radionuclides ^226^Ra, ^232^Th, and ^40^K were analyzed using a Hyper-pure Germanium (HPGe) coaxial detector system Model (ORTEC^®^ GEM40-83-SMP) with serial number 57P51572A, housed in a lead shielding, connected to a multichannel analyzer. Gamma spectrometric measurements were performed following procedures described by Sarker et al. [26]. Each water sample was counted for 50,000 s to obtain gamma spectra with good statistics, and radionuclides were identified based on their characteristic gamma emission energies. The background distribution in the environment around the detector was conducted as described by [27,28]. An empty Marinelli beaker was counted under identical geometry and counting conditions to the samples to obtain the background spectrum. This background was used to correct the net peak area of the targeted radionuclides. These measurements also allowed for the calculations of minimum detectable activities (MDAs) for radionuclides such as ^226^Ra, ^232^Th, and ^40^K, ensuring accurate quantification at low activity levels.

#### 2.3.1. Energy Calibration of the System

Energy calibration assigns each channel of the spectrum to a known gamma-ray energy [29,30]. This was performed by using a CBSS2 multi-nuclide standard source obtained from the Czech Metrology Institute (CMI), which emits gamma rays from 60 keV to 2 MeV. Peak centroids were identified and matched to reference energies and a calibration curve (channel vs. energy) was plotted to confirm linearity and accuracy. Calibration and spectral data acquisition were conducted using an ORTEC^®^ DSPEC-LF processor and analyzed using GammaVision^®^ software, version 8.10.02 [31].

#### 2.3.2. Efficiency Calibration of the System

Efficiency calibration ensures accurate quantification by relating the detector response to gamma-ray energy. The same mixed radionuclide standard was used, and the efficiencies were calculated using Equation (1):

(1) $$\varepsilon_{f}=\frac{N_{c}}{P_{\gamma }\times A_{std}\times T_{std}}$$

where $\varepsilon_{f}$ is the gamma line emission intensity (peak efficiency) (%), *N_c_* is the net counts per second (cps), *P*_γ_ is the gamma-line emission probability of a particular radionuclide, *A_std_* is the decay activity of the standard, and *T_std_* (s) is the counting time of the standard. Activity decay over time was corrected using Equation (2):

(2) $$A_{std\;corected}=A_{std}\times e^{-\lambda t}$$

#### 2.3.3. Activity Concentration Determination

Activities of ^226^Ra, ^232^Th, and ^40^K were derived from high-intensity and low-interference gamma lines: ^214^Pb (295, 351 keV) and ^214^Bi (609 keV) for ^226^Ra, via progeny; ^228^Ac (911, 969 keV) and ^212^Pb (239 keV) for the ^232^Th series; and ^40^K (1460 keV). The specific activity concentrations were calculated using Equation (3) [32]:

(3) $$A_{c}=\frac{N_{c}}{P_{\gamma }\times \varepsilon \times T\times W}$$

where *A_c_* is the activity concentration in the sample in Bq kg^−1^, *N_c_* is the net counts per second (cps), *P*_γ_ is a gamma-line emission probability of a particular radionuclide, *ε* is the gamma line emission intensity (%), *T* (s) is the counting time of the sample, and *W* (kg) is the weight of the sample.

### 2.4. Establishment of Annual Effective Ingestion Dose (AEID) and Total AEID (TAEID)

Annual effective ingestion dose (AEID) is the estimated annual radiation dose, expressed in millisieverts per year (mSv y^−1^), received by an individual through ingestion of radionuclides in drinking water. The total annual effective ingestion dose (TAEID) represents the sum of AEID values for all relevant radionuclides present in a given water source, thereby providing an overall measure of the ingestion-related radiological risk. Both the AEID and TAEID due to the intake of drinking water for three different age groups (infants, children, and adults) were estimated using Equations (4) and (5) [33].

(4) $$AEID\;\left({Svy}^{-1}\right)=A_{C}\times A_{i}\times C_{f}$$

where *A_c_* is the activity concentration of the radionuclide in water (Bq L^−1^), *A_i_* is the annual intake of drinking water (L y^−1^), and *C_f_* is the ingested dose conversion factor for each radionuclide (Sv Bq^−1^).

(5) $$TAEID\;\left({Svy}^{-1}\right)=\sum_{i}^{n}A_{c}\times A_{i}\times C_{f}$$

The values of the *A_i_* and *C_f_* vary with both the radionuclide and the age of individuals ingesting the radionuclide, as shown in Table 1 [1,34,35,36].

The somatic as well as hereditary effects on organs have also been estimated using the lifetime fatal cancer risks and hereditary effects coefficient, calculated by Equations (6)–(9) [37,38,39]:

(6) $$FCR\;=TAEID\times {RF}_{c}$$

where fatal cancer risk (*FCR*) is the estimated probability that an individual will die from cancer as a result of a specific radiation exposure over their lifetime.

(7) $$LFCR=TAEID\times A_{g}\times {RF}_{c}$$

where the lifetime fatal cancer risk (*LCFR*) quantifies the probability that an individual will develop and die from cancer over their expected lifespan due to chronic ingestion of radionuclide-contaminated water [1].

(8) $$SHE=TAEID\times {HEF}_{c}\;\;\;\;$$

where severe hereditary effect (*SHE*) denotes the probabilistic health outcomes from radiation exposure, including cancer and heritable effects and *HEF_c_* denotes the hereditary effect coefficients (0.2 × 10^−1^ Sv^−1^) for the general public, assumed to be within the age of 70 years, i.e., a lifetime of exposure to low-level radiation.

(9) $$ELHE=TAEID\times A_{g}\times {HEF}_{c}\;\;\;$$

where excess lifetime hereditary effect (*ELHE*) denotes the additional probability of heritable genetic effects occurring in offspring due to parental exposure. The *A_g_*, is the adult lifetime age in years, 68 years for females and 64 years for males [40].

### 2.5. Statistical Analysis

Multivariate statistical analysis was used to investigate the relationship among the variables using IBM SPSS, version 25 [41]. Pearson’s correlation coefficients (r) were computed to examine the linear relationships between radionuclide concentrations, ingestion dose metrics, and water quality parameters (e.g., pH, electrical conductivity (EC), and total dissolved solids (TDS)) [42]. One-way Analysis of Variance (ANOVA) was applied to test for significant differences in mean radionuclide concentrations and risk indices across sampling locations.

## 3. Results and Discussions

### 3.1. Radionuclide Activity Concentration

Table 2 presents the activity concentration of ^226^Ra and ^40^K, together with the measured water quality parameters (pH, EC, and TDS), from water samples collected at 17 sampling locations around the Geita Gold Mine area.

Although ^232^Th was analyzed in this study, its activity was below the minimum detectable activity (MDA) in all samples. Likewise, ^212^Pb was not detected. The 239 keV peak remained below the sample-specific MDA under the applied counting time. In practice, detecting ^212^Pb in water by gamma spectrometry is difficult due to its short half-life and low-level activity (<0.1 Bq L^−1^) [43,44]. Thorium is highly insoluble, tends to bind to solid particles, and is typically present at very low levels in groundwater due to its association with mineral phases, making it often undetectable, even near ore bodies [45]. Accordingly, the results and discussion in this paper are limited to the distribution and potential effects of the radionuclide concentrations of ^226^Ra and ^40^K in water samples from the Geita Gold Mine area.

The results, in Table 2, show a substantial spatial variability of the parameters among samples. The activity concentrations of ^226^Ra in water samples ranged from 14 to 130 mBq L^−1^, with an average of 57 ± 32 mBq L^−1^. The maximum concentration of ^226^Ra in water samples was observed at location MW01 (130 mBq L^−1^), adjacent to the tailings storage facility. This elevated ^226^Ra concentration aligns with findings from water samples taken from other tailings facilities near gold mines. For instance, studies at the Mary Kathleen mine in Australia has reported higher ^226^Ra concentration in adjacent water bodies due to seepage from tailings dams [46]. These findings underscore the importance of effective tailings management to mitigate radiological risks to surrounding communities. In contrast, the lowest concentration of ^226^Ra was recorded at location MW05 (14 mBq L^−1^). Overall, the observed ^226^Ra concentrations of all the samples are significantly below the IAEA drinking water recommended level of 1000 mBq L^−1^ [47] suggesting that the radiological risks from this radionuclide are currently with safe limits.

The concentration of ^40^K ranged from 2370 to 7880 mBq L^−1^, averaging 5026 ± 1787 mBq L^−1^. Elevated ^40^K in samples such as MW03 and MW10 are likely due to the local geology, which is known to contain potassium-bearing minerals [21,48], consistent with findings from other gold Archean terrains. The overall results suggest that water may not pose significant radiological risks to the population under the current conditions.

Compared to the results from studies from other regions (Table 3), the average level of ^226^Ra in Geita Gold Mine water (57 mBq L^−1^) is substantially lower than values reported for water in the Kilowoko River at the Mkuju River Project (2500 mBq L^−1^) in Tanzania [12]. The activity concentration is approximately 39%, 52%, 80%, and 85% lower than the average values obtained for dug wells in Yemen [49], tap water in Iraq [50], wells in Egypt [51], and bottled water in Turkey [35], respectively. These values are significantly below the WHO guidance limit (1000 mBq L^−1^) [52]. On the other hand, significantly elevated levels were observed in several groundwater sources in Nigeria [53,54] and Yemen [55], and in drinking water in Iraq [56] (Table 3).

The average activity concentration of ^40^K (5026 mBq L^−1^) is 15%, 44%, 67%, and 78% lower than the values reported in Turkish bottled mineral water [35], Tanzanian swamp water [11], Egyptian wells [51] and Iraqi tap water [50], respectively. By contrast, this average value exceeded the values reported for Nigerian private dug wells [54], Iraqi drinking water [56], and Yemeni ground and spring water [55]. These comparisons highlight that, although Geita’s NORM levels are moderate by global standards, local factors such as underlying bedrock formation, mining practices, and ground chemistry drive substantial spatial heterogeneity. As such, continued monitoring against WHO and IAEA guidelines, particularly in regions with documented elevated NORM seepage from mining residues, should be undertaken.

### 3.2. Water Quality Parameters

The water quality parameters varied across sampling locations (Table 2). The pH values ranged from 6.15 to 8.00 with an average of 7.23 ± 0.40, suggesting near-neutral to slightly alkaline conditions, which generally favors radium desorption from sediments into aqueous phase. The samples analyzed were within the WHO’s admissible limit (6.5–8.5) for drinking water [47]. These pH values observed are slightly lower than those observed in Cameroon [57] and slightly higher than those observed in East Cameroon [58].

The electrical conductivity (EC) values ranged from 40 µS cm^−1^ to 2070 µS cm^−1^, with an average of 597 ± 746 µS cm^−1^. The average value was lower than the WHO’s admissible value of 1400 µS cm^−1^ [47,58]. The average value was slightly higher than the value of 518 µS cm^−1^ observed at the Asikam gold mining community in Ghana [59] and Cameroon [60]. However, higher value of 2070 µS cm^−1^ at MW01, 2040 µS cm^−1^ at MW02 and 2060 µS cm^−1^ at MW03 were observed, which could be an indicator of presence of contaminants such as sodium, potassium, chlorides, and sulfates [61].

TDS ranged from 20 mg L^−1^ to 1330 mg L^−1^, with an average of 397 ± 485 mg L^−1^, which is slightly below the palatability level of the WHO-recommended value of 600 mg L^−1^ [47]. It was further observed that the TDS values for water from the surrounding community were also below the WHO-recommended value of 600 mg L^−1^ [48]. The TDS values at some locations in the mining areas, especially MW01–MW03, were observed to be slightly greater than the WHO-recommended unpalatability level of 1000 mg L^−1^ [47]. According to the WHO, there is no health-based limit for TDS in drinking water, as TDS in drinking water at a concentration well below toxic effects may occur [47]. Although at some locations the measured water quality parameters, including pH (6.15 to 8.00), EC (40 to 2070 µS cm^−1^), and TDS (20 to 1330 mg L^−1^), were below the levels recommended by the WHO, it is advisable to conduct ongoing monitoring to identify any potential alterations that may arise in the future due to mining activities.

### 3.3. Annual Effective Ingestion Dose (AEID) and Total Annual Effective Ingestion Dose (TAEID)

The estimated annual effective ingestion dose (AEID) of ^226^Ra and ^40^K as well as the total annual effective ingestion doses (TAEID) across different age groups: infants (0^−1^ y), children (12–17 y), and adults (>17) are presented in Table 4. The values of the AEID for ^226^Ra ranged from 9.9 to 91.7 µSv y^−1^ (mean 40.2 ± 22.6 µSv y^−1^) for infants; from 3.9 to 36.4 µSv y^−1^ (mean 16.0 ± 9.0 µSv y^−1^) for children and from 2.9 to 27.3 µSv y^−1^ (mean 12.0 ± 6.7 µSv y^−1^) for adults. For ^40^K, the ranges of the AEID were 22.0–73.3 µSv y^−1^ (mean 46.7 ± 16.6 µSv y^−1^) for infants; 10.8–35.9 µSv y^−1^ (mean 22.9 ± 8.1 µSv y^−1^) for children; and 11.0–36.6 µSv y^−1^ (mean 23.4 ± 8.3 µSv y^−1^) for adults. Notably, compared to other locations, the water samples from locations MW01 and MW03 exhibited the highest AEID values of ^226^Ra and ^40^K, respectively, for all age groups. The samples were collected directly from the water of the tailings storage facility, indicating that the elevated dose is a result of accumulation and concentration within the impoundment itself.

For the total annual effective ingestion dose (TAEID), the doses obtained exhibited clear age-dependency. The doses ranged from 35.1 to 148.7 µSv y^−1^ (mean 87.0 ± 31.5 µSv y^−1^) for infants; from 16.3 to 64.3 µSv y^−1^ (mean 38.8 ± 13.7 µSv y^−1^) for children and from 15.5 to 56.8 µSv y^−1^ (mean 35.4 ± 12.0 µSv y^−1^) for adults (Table 4). The TAEID values for all age groups across all sites were below the WHO’s age-specific guidance thresholds of 260 µSv y^−1^ for infants, 200 µSv y^−1^ for children, and 100 µSv y^−1^ for adults [6,52]. Additionally, the TAEID values were below the IAEA’s reference level of 1000 µSv y^−1^ for drinking water [9,62] exposure. The TAEID values were also below the ICRP’s limit of 100 µSv y^−1^ from a single source [3,63], except for the values for infants from samples collected at the tailings-adjacent locations MW01 (148.7 µSv y^−1^) and MW03 (141.0 µSv y^−1^). These exceedances highlight the importance of targeted monitoring and the implementation of mitigation strategies, such as engineered seepage barriers, tailings liners, and diversion systems to protect downstream communities, especially those that may rely on nearby groundwater sources for domestic use.

Moreover, it was noted that infants received the highest averages of AEID and TAEID compared to other age groups. This may be due to their greater water intake per unit body mass and enhanced accumulation of ^226^Ra in developing bone tissue [64]. These findings are consistent with the studies conducted in Malaysia [65], Turkey [66], and Pakistan [67], where infants were also found to receive the highest ingestion doses. Although infants exhibit the highest absolute ingestion doses, children are considered physiologically more vulnerable due to their rapid skeletal growth. This highlights the need for precautionary measures to minimize the intake of radionuclide-contaminated water [64] during childhood, in order to reduce the long term risks of developing born sarcomas [68]. As such, continuous radiological monitoring of NORM should be conducted.

Conversely, for all age groups, the lowest levels of AEID were observed from samples collected at MW05 for ^226^Ra and MW04 for ^40^K. Additionally, for all age groups, the lowest levels of TAEID were observed from samples collected at MW05. These levels likely reflect baseline background conditions, with minimal anthropogenic input. This is consistent with studies near non-contaminated sites or upstream of mining areas, where TAEID values for infants typically range between 20 and 50 µSv y^−1^. For example, in Baling, Malaysia, Ahmad et al. [65] reported similar low-dose estimates in spring waters unaffected by industrial discharge. Likewise, Kimaro and Mohammed [11] found comparable low NORM levels in Tanzanian spring waters not influenced by uranium extraction activities.

### 3.4. Cancer Risks and Hereditary Effects

The estimated radiological health risks due to ingestion of water contaminated with ^226^Ra and ^40^K around the Geita Gold Mine were evaluated as shown in Table 5. The fatal cancer risk (FCR) ranged from 0.9 × 10^−6^ to 3.1 × 10^−6^, with a mean value of (2 ± 0.7) × 10^−6^, suggesting generally low but variable risk levels. This means that at least 2 people out of 1,000,000 would develop fatal cancer from long-term ingestion of water.

The LFCR ranged from 0.5 × 10^–4^ to 2.1 × 10^–4^ with an average of (1.3 ± 0.4) × 10^–4^ for males and from 0.6 × 10^−4^ to 2.2 × 10^−4^ with an average of (1.3 ± 0.5) × 10^−4^ for females, as shown in Table 5. The LFCR values were higher where ^226^Ra is elevated, near tailings, and where the bedrock is rich in K-bearing feldspar, whereas water quality parameters (pH, EC, and TDS) only slightly influenced LCFR values within the observed ranges. On average, this translates to approximately one additional fatal cancer case per 10,000 consumers of water, attributed to ingestion of ^226^Ra and ^40^K. Importantly, all observed LCFR values are below the ICRP’s guidance level of 5.0 × 10^–4^ [63], indicating that lifetime risks in the study area are within acceptable limits under current conditions. However, the upper-range values of sites MW01 and MW03, closest to the tailings facility, are about 40% of the ICRP’s guidance level, underscoring the need for targeted mitigation measures and continued surveillance in areas with potential radionuclide seepage.

The estimated SHE ranged from 3.1 × 10^–8^ to 11.4 × 10^–8^, with an average of (7.1 ± 2.4) × 10^–8^ as shown in Table 5. This corresponds to roughly 7 out of 100 million people potentially suffering hereditary effects from drinking water around the Geita Gold Mine. The low SHE may be attributed to the low source terms (^226^Ra and ^40^K), which translates into smaller gonadal doses and hence smaller hereditary risk estimates [1].

The excess lifetime hereditary effect (ELHE) index, which reflects cumulative genetic risk over a lifetime, ranged from 2.0 × 10^–6^ to 7.3 × 10^–6^ with an average of (4.5 ± 1.5) × 10^–6^ for males and 2.1 × 10^–6^ to 7.7 × 10^–6^ with an average of (4.8 ± 1.6) × 10^–6^ for females (Table 5).

This indicates that out of 1,000,000 individuals chronically ingesting the water, approximately two to eight cases of heritable genetic effects might occur over a lifetime. The marginally higher ELHE in females reflects sex-specific physiological and metabolic differences and the greater radiosensitivity of female-specific tissues (e.g., breast, thyroid, and gonads) that enhance radionuclide retention, coupled with longer female life expectancy, which extends the period during which hereditary effects can manifest [8,69]. Females typically have a longer life expectancy than males, thereby increasing the duration over which radiation-induced malignancies may develop. Additionally, the long-term radiosensitivity in females, specifically in the breasts, thyroid, and reproductive organs, is higher than that in males [34,70].

### 3.5. Comparison of Cancer Risks and Hereditary Effects with Other Regions

Table 6 compares the average fatal cancer risk (FCR), lifetime fatal cancer risk (LFCR), severe hereditary effect (SHE), and excess lifetime hereditary effect (ELHE) for water sources around the Geita Gold Mine area to other regions. In Geita, the mean values of FCR (2.0 × 10^−6^), LFCR (1.3 × 10^−4^), SHE (7.1 × 10^−8^), and ELHE (4.8 × 10^−6^) are significantly lower than other mean values reported, reflecting minimal genetic risk. By contrast, Nigerian waters from Delta State [71] exhibit an FCR of up to 1.9 × 10^−4^, an LFCR of up to 1.3 × 10^−2^, and SHE (6.8 × 10^−6^). However, the ELHE value at Geita aligned with the results for Nigeria borehole water.

Spring water in South Africa’s goldfields [39] also shows elevated values of FCR (1.8 × 10^−4^), LFCR (1.2 × 10^−2^), SHE (6.4 × 10^−6^), and ELHE (4.5 × 10^−4^), consistent with uranium-bearing and granites in that region. Similarly, phosphate-contaminated waters in Egypt [72] reported FCR (5.6 × 10^−5^), LFCR (4.0 × 10^−3^), SHE (2.0 × 10^−6^), and ELHE (1.4 × 10^−4^), reflecting industrial waste inputs. Conversely, Ghanaian bottled and borehole waters yield negligible risk metrics, with the lowest FCR down to 3.2 × 10^−9^ and ELHE down to 6.9 × 10^−11^, which is attributed to aquifer isolation and low background radioactivity [73,74]. These comparatively lower values at Geita are likely influenced by the geological characteristics of the area. Specifically, the mineralogical composition of the underlying rocks in the Geita region is known to contain relatively lower concentrations of uranium and thorium-bearing minerals compared to other mining belts in Africa.

**Table 6:** Comparison of radiological risk indices (FCR, LFCR, SHE, ELHE) for water sources in the Geita Gold Mine area and other selected regions.

Country	Water Type	FCR	LFCR	SHE	ELHE	Reference
Tanzania	Spring, rivers and ponds	2.0 × 10^−6^	1.3 × 10^−4^	7.1 × 10^−8^	4.8 × 10^−6^	This study
Nigeria	Borehole	1.9 × 10^−4^	1.3 × 10^−2^	6.8 × 10^−6^	4.8 × 10^−6^	[71]
Nigeria	Salt lake	6.3 × 10^−7^	4.4 × 10^−5^	2.3 × 10^−8^	2.7× 10^−7^	[37]
Nigeria	Pond	2.2 × 10^−5^	1.5 × 10^−3^	2.3 × 10^−8^	2.7× 10^−7^	[75]
Nigeria	Rivers	1.5 × 10^−5^	1.1 × 10^−3^	5.6 × 10^−7^	3.9× 10^−4^	[75]
South Africa	Natural spring bottled drinking water	1.8 × 10^−4^	1.2 × 10^−2^	6.4 × 10^−6^	4.5 × 10^−4^	[39]
Egypt	Water from phosphate-polluted area	5.6 × 10^−5^	4.0 × 10^−3^	2.0 × 10^−6^	1.4 × 10^−4^	[72]
Ghana	Bottled drinking water	7.3 × 10^−7^	-	1.8 × 10^−8^	-	[73]
	Spring borehole and well water	3.2 × 10^−9^		6.9 × 10^−11^		[74]

All the calculated FCR and LFCR values fall below the United States Environmental Protection Agency (USEPA)’s acceptable cancer fatality risk limits of 1.0 × 10^−6^ to 1.0 × 10^−4^ (i.e., 1 person out of 1,000,000 to 1 person out of 10,000 persons suffering from some form of cancer fatality).

### 3.6. Spatial Variability of Radionuclide Activity, Radiological Risk Indices and Water Parameters

A one-way ANOVA revealed highly significant spatial differences in radionuclide activities and ingestion-related health risk indices in the 51 samples across the 17 sampling sites. The three (3) variables ^226^Ra, ^40^K, and EC exhibited extremely significant differences among the 17 sampling locations (*p* < 0.001). The very high F-values (55.67 for ^226^Ra; 34.02 for ^40^K, and 196.58 for EC) confirm that site identity (i.e., proximity to tailings and local geology) governs their variability.

The adjusted R^2^ values approach unity (≥0.914), indicating that over 91% of the variability in radionuclide concentrations and virtually 100% of the variability in EC is attributable to site factors alone. The large F-statistic for EC underscores its role as an integrative indicator of mining-related inputs (process water, tailings leachate) that mobilize NORM. EC’s near-perfect R^2^ (0.999) suggests that ionic strength fluctuates in lockstep with localized contamination sources.

The high R^2^ and F-values suggest a site-specific monitoring system that focuses on known hotspots, such as tailings storage facilities, where EC and radionuclide levels were observed to be high. Given EC’s strong explanatory power, regular EC measurements could be used as a quick way to find places that need more detailed spectrometry. Being aware of sharp spatial contrasts in space can also help with remediation activities (such as seepage barriers) at places that are more affected, while also making sure that off-pit village water stays within acceptable limits.

Post hoc Tukey’s HSD identified MW01 and MW03 as significantly elevated (*p* < 0.001) relative to other sites for both radionuclide concentration and dose metrics. Both sites are located at the tailings storage facilities and known seepage zones, supporting the inference of tailings-derived contamination. This pattern aligns with seepage dynamics reported near Ghana’s Chirano Gold Mine [76], further emphasizing the need for site-specific radiological monitoring near mining infrastructure.

Water parameters showed contrasting patterns. Electrical conductivity (EC) varied largely between sites (F_16_,_34_ = 196.58, *p* < 0.001), whereas pH (mean ≈ 7.23) and TDS (mean ≈ 397 mg L^−1^) remained statistically uniform. The strong spatial variability in EC, an integrative measure of ionic strength, likely reflects differential inputs of mining process waters and tailings seepage that enhance radionuclide mobilization. Similarly, EC-driven radionuclide transport documented in South African goldfields showed that EC anomalies correlated with ^226^Ra hotspots near tailings facilities.

Fatal cancer risk (FCR), severe hereditary effect (SHE), lifetime fatal cancer risks (LFCR), and excess lifetime hereditary effect (ELHE) values likewise exhibited significant between-group differences (all F > 27, *p* < 0.001), confirming pronounced spatial heterogeneity in both source terms and health risks indices, likely driven by proximity to mine tailings storage facilities and geological variability [77].

### 3.7. Correlation Analysis

The correlation results (Table 7) show that the ingestion dose and the associated cancer and hereditary risks are largely driven by the ^226^Ra and ^40^K. ^226^Ra showed a moderate positive correlation with infant TAEID (*r* = 0.653, *p* < 0.01), child TAEID (*r* = 0.653, *p* < 0.01), and all risk indices, FCR (*r* = 0.653, *p* < 0.01), LFCR (*r* = 0.997, *p* < 0.01), SHE (*r* = 0.507, *p* < 0.01), and ELHE (*r* = 0.662, *p* < 0.01). ^40^K followed a similar trend, showing strong correlations with all ingestion dose values (TAEID: *r* = 0.872, *p* < 0.01) and risk indices (FCR: *r* = 0.871, *p* < 0.01; LFCR: *r* = 0.865, *p* < 0.01; SHE: *r* = 0.870, *p* < 0.01; ELHE: *r* = 0.865, *p* < 0.01). This pattern clearly indicates that variations in these two radionuclides have a direct influence on age-specific effective doses and the resulting health risk estimates.

Water quality parameters showed more varied relationships. EC was strongly linked to TDS (*r* = 0.843, *p* < 0.01) and moderately associated with pH (*r* = 0.297, *p* < 0.05), but its connection to radionuclide activity was weak and not statistically significant for ^226^Ra (*r* = 0.080, *p* > 0.05) or ^40^K (*r* = −0.065, *p* > 0.05). Even so, EC’s broader role as an indicator of ionic strength means it remains relevant for understanding NORM mobility.

pH showed moderate positive correlations with ^226^Ra (*r* = 0.320, *p* < 0.05) and ^40^K (*r* = 0.496, *p* < 0.01), suggesting that pH-dependent adsorption and desorption processes may influence their concentrations. TDS, on the other hand, was not significantly associated with radionuclide activities or most dose metrics, apart from its strong correlation with EC. Similar trends were reported for natural spring bottled drinking water from South Africa [39] and the Elba Protective area, Egypt [64].

The relationships between TAEID and the various risk indices were extremely high (FCR: r = 0.997, LFCR: r = 0.999, SHE: r = 0.987, ELHE: r = 0.999; all *p* < 0.01), reflecting their shared basis in dose–risk conversion. Similarly, the risk indices themselves were almost perfectly correlated with each other (≥0.986, *p* < 0.01), underscoring the internal consistency of the risk assessment.

These results reinforce that ^226^Ra and ^40^K are the main contributors to ingestion-related radiological risk in the study area, while water chemistry parameters such as pH and EC play supporting roles, and TDS has limited predictive value within the narrow range observed.

## 4. Conclusions

The assessment of naturally occurring radioactive materials in water around the Geita Gold Mine was conducted. The results indicated clear spatial heterogeneity driven by mining operations. Activity concentrations of ^226^Ra (14 to 130 mBq L^−1^, mean 57 ± 32 mBq L^−1^) and ^40^K (2370 to 7880 mBq L^−1^, mean 5026 ± 1787 mBq L^−1^) remain well below the WHO and IAEA’s guidance values for drinking water, but localized concentration “hotspots” adjacent to the tailings facility (MW01 and MW03) exhibit significantly elevated level. In contrast, ^232^Th was below minimum detection limits, likely due to its low solubility under the existing pH and redox conditions.

In addition, the age-stratified dose modeling results revealed that infants receive high values of TAEID (35.1–148.7 µSv y^−1^), followed by children (16.3–64.3 µSv y^−1^), and adults (15.5–56.8 µSv y^−1^). Although the average estimated doses for all groups fall below the WHO’s age-specific limits of 260, 200, 100 µSv y^−1^ for infants, children, and adults, respectively, two tailings-adjacent sites, MW01 (148.7 µSv y^−1^) and MW03 (141.0 µSv y^−1^), exceeded the ICRP’s infant threshold of 100 µSv y^−1^, underscoring the need for targeted mitigation.

Cancer-risk estimates FCR (0.9 × 10^−6^–3.1 × 10^−6^), LFCR (0.5 × 10^−4^–2.2 × 10^−4^) and hereditary effects SHE (3.1 × 10^−8^–11.4 × 10^−8^) and ELHE (2.0 × 10^−6^–7.7 × 10^−6^) likewise remain below international reference levels, with slightly higher values in females reflecting longer life expectancy and greater radiosensitivity of female-specific tissues. This study thus provides a robust, age-stratified baseline of ingestion doses and health-risk indices, filling a critical gap for gold mining regions in Tanzania and offering a template for similar assessments elsewhere.

## Figures and Tables

**Table 1:** Dose conversion factors and annual water intake.

Age Group (y)	Dose Conversion Factor (Sv Bq^−1^)		Annual Water Intake (L y^−1^)
	^226^Ra	^40^K	
<1	4.7 × 10^−6^	6.2 × 10^−8^	150
7–12	8.0 × 10^−7^	1.3 × 10^−8^	350
>17	2.8 × 10^−7^	6.2 × 10^−9^	730

**Table 2:** Activity concentration of ^226^Ra and ^40^K and water quality parameters.

Sample ID and Location	UTM Northings	UTM Eastings	Activity Concentration		Water Quality Parameter		
			^226^Ra (mBq L^−1^)	^40^K (mBq L^−1^)	pH	TDS (mg L^−1^)	EC (µS cm^−1^)
MW01 (3), Mining area	408,530	968,5973	130	6130	8.00	1330	2070
MW02 (3), Mining area	408,530	9,685,973	28	7230	7.26	1300	2040
MW03 (3), Mining area	408,530	9,685,973	96	7880	7.71	1320	2060
MW04 (3), Mining area	408,530	9,685,973	25	2370	7.45	660	1020
MW05 (3), Mining area	409,253	9,681,623	14	2710	7.09	530	840
MW06 (3), Mining area	409,146	9,681,936	20	2500	6.15	350	600
MW07 (3), Mining area	408,663	9,681,412	60	3100	7.06	120	190
MW08 (3), Mining area	408,582	9,681,719	48	6920	7.45	140	220
MW09 (3), Mining area	396,385	9,681,250	32	6110	7.59	60	100
MW10 (3), Mining area	396,385	9,681,397	19	7260	7.14	70	40
NKW1 (3), Nyakabale village	407,222	9,684,202	70	3890	6.95	70	110
NKW2 (3), Nyakabale village	407,302	9,683,900	67	3890	6.89	590	150
NKW3 (3), Nyakabale village	405,135	9,685,853	80	4230	7.31	40	50
MGW1 (3), Mgusu village	395,693	9,683,748	50	5340	7.19	30	50
MGW2 (3), Mgusu village	395,713	9,683,639	72	4440	7.40	30	490
MCW1 (3), Machinjioni village	394,428	9,684,716	92	5740	7.16	20	40
MBW1 (3), Mabubi River	395,088	9,684,266	67	5710	7.16	90	130
Average ± SD ^a^			57 ± 32	5026 ± 1787	7.23 ± 0.4	397 ± 485	597 ± 746
Range			14–130	2370–7880	6.15–8	20–1330	40–2070

The values in brackets indicate the number of samples analyzed. ^a^ Denotes standard deviation.

**Table 3:** Activity concentrations of ^226^Ra and ^40^K in water sources from different countries.

Source/Type of Water	Country	Activity Concentration (mBq L^−1^)		References
		^226^Ra	^40^K	
Tailings, spring water and ponds	Tanzania	57	5026	This study
River water (Kilowoko River)	Tanzania	2500	11,000	[12]
Spring water	Tanzania	-	2820	[11]
Bottled mineral water	Turkey	380	4260	[35]
Tap water	Iraq	121	1091	[50]
Wells	Egypt	270	1610	[51]
Groundwater	Yemen	2950	34,900	[55]
Spring water	Yemen	3480	16,500	[55]
Dug wells	Yemen	94	3306	[49]
Dug and drilled a well for water	Nigeria	4540	2940	[53]
Private dug wells	Nigeria	7150	13,540	[54]
Drinking water	Iraq	76,000	447,000	[56]
Guidance level		1000	-	[47]

**Table 4:** Annual effective ingestion dose (AEID) of ^226^Ra and ^40^K, and total AEID (TAEID) for different age groups.

Sample Code	^226^Ra-AEID (µSv y^−1^)			^40^K-AEID (µSv y^−1^)			TAEID (µSv y^−1^)		
	Infants	Children	Adults	Infants	Children	Adults	Infants	Children	Adults
MW01 (3)	91.7	36.4	27.3	57.0	27.9	28.5	148.7	64.3	55.8
MW02 (3)	19.7	7.8	5.9	67.2	32.9	33.6	87.0	40.7	39.5
MW03 (3)	67.7	26.9	20.2	73.3	35.9	36.6	141.0	62.7	56.8
MW04 (3)	17.6	7.0	5.3	22.0	10.8	11.0	39.7	17.8	16.3
MW05 (3)	9.9	3.9	2.9	25.2	12.3	12.6	35.1	16.3	15.5
MW06 (3)	14.1	5.6	4.2	23.3	11.4	11.6	37.4	17.0	15.8
MW07 (3)	42.3	16.8	12.6	28.8	14.1	14.4	71.1	30.9	27.0
MW08 (3)	33.8	13.4	10.1	64.4	31.5	32.2	98.2	44.9	42.3
MW09 (3)	22.6	9.0	6.7	56.8	27.8	28.4	79.4	36.8	35.1
MW10 (3)	13.4	5.3	4.0	67.5	33.0	33.8	80.9	38.4	37.8
NKW1 (3)	49.4	19.6	14.7	36.2	17.7	18.1	85.5	37.3	32.8
NKW2 (3)	47.2	18.8	14.1	36.2	17.7	18.1	83.4	36.5	32.2
NKW3 (3)	56.4	22.4	16.8	39.3	19.3	19.7	95.7	41.7	36.5
MGW1 (3)	35.3	14.0	10.5	49.7	24.3	24.8	84.9	38.3	35.3
MGW2 (3)	50.8	20.2	15.1	41.3	20.2	20.7	92.1	40.4	35.8
MCW1 (3)	64.9	25.8	19.3	53.4	26.1	26.7	118.2	51.9	46.0
MBW1 (3)	47.2	18.8	14.1	53.1	26.0	26.6	100.3	44.7	40.6
Mean ± SD ^a^	40.2 ± 22.6	16.0 ± 9.0	12.0 ± 6.7	46.7 ± 16.6	22.9 ± 8.1	23.4 ± 8.3	87.0 ± 31.5	38.8 ± 13.7	35.4 ± 12.0
Range	9.9–91.7	3.9–36.4	2.9–27.3	22.0–73.3	10.8–35.9	11.0–36.6	35.1–148.7	16.3–64.3	15.5–56.8

^a^ Denotes standard deviation.

**Table 5:** Cancer risk and hereditary effect on individuals due to ingestion of ^226^Ra and ^40^K in drinking water.

Sample ID	FCR × 10^−6^	LFCR × 10^–4^		SHE × 10^−8^	ELHE × 10^–6^	
		Male	Female		Male	Female
MW01 (3)	3.1	2.1	2.2	11.2	7.1	7.6
MW02 (3)	2.2	1.4	1.5	7.9	5.1	5.4
MW03 (3)	3.1	2.0	2.1	11.4	7.3	7.7
MW04 (3)	0.9	0.6	0.6	3.3	2.1	2.2
MW05 (3)	0.9	0.5	0.6	3.1	2.0	2.1
MW06 (3)	0.9	0.6	0.6	3.2	2.0	2.2
MW07 (3)	1.5	1.0	1.1	5.4	3.5	3.7
MWS08 (3)	2.3	1.5	1.6	8.5	5.4	5.8
MW09 (3)	2.0	1.2	1.3	7.0	4.5	4.8
MW10 (3)	2.1	1.3	1.4	7.6	4.8	5.1
NKW1 (3)	1.8	1.2	1.2	6.6	4.2	4.5
NKW2 (3)	1.8	1.1	1.2	6.4	4.1	4.4
NKW3 (3)	2.0	1.3	1.4	7.3	4.7	5.0
MGW1 (3)	2.0	1.2	1.3	7.1	4.5	4.8
MGW2 (3)	2.0	1.3	1.3	7.2	4.6	4.9
MCW1 (3)	2.5	1.6	1.7	9.2	5.9	6.3
MBW1 (3)	2.2	1.4	1.5	8.1	5.2	5.5
Mean ± SD ^a^	2.0 ± 0.7	1.3 ± 0.4	1.3 ± 0.5	7.1 ± 2.4	4.5 ± 1.5	4.8 ± 1.6
Range	0.9–3.1	0.5–2.1	0.6–2.2	3.1–11.4	2.0–7.3	2.1–7.7

^a^ Denotes standard deviation.

**Table 7:** Pearson correlation coefficients between activities, hydrochemical parameters, ingestion doses, and radiological risk for 51 water samples from 17 location around Geita Gold Mine area.

Parameter	^226^Ra	^40^K	pH	TDS	EC	TAEID	FCR	LCFR	SHE	ELHE
Ra-226	1.000									
K-40	0.490 **	1.000								
pH	0.320 *	0.496 **	1.000							
TDS	−0.147	0.025	0.103	1.000						
EC	0.080	−0.065	0.297 *	0.843 **	1.000					
TAEID	0.653 **	0.872 **	0.546 **	−0.015	0.007	1.000				
FCR	0.653 **	0.871 **	0.540 **	−0.002	0.026	0.997 **	1.000			
LFCR	0.997 **	0.865 **	0.544 **	−0.009	0.017	0.999 **	0.998 **	1.000		
SHE (Adults)	0.507 **	0.870 **	0.546 **	−0.005	0.011	0.987 **	0.987 **	0.987 **	1.000	
ELHE (Adults)	0.662 **	0.865 **	0.544 **	−0.010	0.016	0.999 **	0.998 **	1.000 **	0.986 **	1.000

* Correlation is significant at the 0.05 level (2-tailed). ** Correlation is significant at the 0.01 level (2-tailed).

## Data Availability

The original contributions presented in this study are included in the article. Further inquiries can be directed to the corresponding author.
